# Macrophage Resistance to HIV-1 Infection Is Enhanced by the Neuropeptides VIP and PACAP

**DOI:** 10.1371/journal.pone.0067701

**Published:** 2013-06-20

**Authors:** Jairo R. Temerozo, Rafael Joaquim, Eduardo G. Regis, Wilson Savino, Dumith Chequer Bou-Habib

**Affiliations:** 1 Laboratory on Thymus Research, Oswaldo Cruz Institute/Fiocruz, Rio de Janeiro, Brazil; 2 Laboratory of Microbiology, Central Hospital of Maputo, Maputo, Mozambique; University of Nebraska Medical Center, United States of America

## Abstract

It is well established that host factors can modulate HIV-1 replication in macrophages, critical cells in the pathogenesis of HIV-1 infection due to their ability to continuously produce virus. The neuropeptides VIP and PACAP induce well-characterized effects on macrophages through binding to the G protein-coupled receptors VPAC1, VPAC2 and PAC1, but their influence on HIV-1 production by these cells has not been established. Here, we describe that VIP and PACAP reduce macrophage production of HIV-1, acting in a synergistic or additive manner to decrease viral growth. Using receptor antagonists, we detected that the HIV-1 inhibition promoted by VIP is dependent on its ligation to VPAC1/2, whereas PACAP decreases HIV-1 growth via activation of the VPAC1/2 and PAC1 receptors. Specific agonists of VPAC2 or PAC1 decrease macrophage production of HIV-1, whereas sole activation of VPAC1 enhances viral growth. However, the combination of specific agonists mimicking the receptor preference of the natural neuropeptides reproduces the ability of VIP and PACAP to increase macrophage resistance to HIV-1 replication. VIP and PACAP up-regulated macrophage secretion of the β-chemokines CCL3 and CCL5 and the cytokine IL-10, whose neutralization reversed the neuropeptide-induced inhibition of HIV-1 replication. Our results suggest that VIP and PACAP and the receptors VPAC2 and PAC1 could be used as targets for developing alternative therapeutic strategies for HIV-1 infection.

## Introduction

The neuropeptides Vasoactive Intestinal Peptide (VIP) and Pituitary Adenylate Cyclase-activating Peptide (PACAP) belong to the secretin/glucagon family of peptides and were initially discovered due to their vasodilatation properties on the gastrointestinal tract and ability to activate rat pituitary adenylate cyclase, respectively [1], [2]. VIP and PACAP present a 68% homology in their amino acid sequences, and share many biological properties [3], [4] through their interaction with the G protein-coupled receptors VPAC1, VPAC2 and PAC1. PACAP binds to all three receptors, with higher affinity to PAC1, while VIP interacts preferentially with VPAC1 and VPAC2 [5]–[8]. VIP and PACAP are produced by Th2 CD4^+^ and CD8^+^ T cells, and their receptors are expressed by a variety of cell types, including T cells, macrophages and dendritic cells [4].

VIP and PACAP have well-characterized effects on the immune system and anti-inflammatory properties, including inhibition of macrophage adherence and down-regulation of inflammatory cytokines and reactive oxygen species [9], [10]–[14]. Moreover, they can induce production of the anti-inflammatory cytokine IL-10 [12], [14]. Due to their immunomodulatory properties, both neuropeptides have been considered as promising therapeutic agents for a range of pathologies [15]–[17].

Macrophages play a central role in the pathogenesis of the human immunodeficiency virus type 1 (HIV-1) infection due to their ability to resist HIV-1-mediated cytopathic effects and to continuously produce virus even in the presence of antiretrovirals [18]–[20]. They function as an HIV-1 reservoir and contribute in HIV-1 transmission to CD4^+^ T cells and virus propagation in lymphoid tissues [21], [22]. Considering that HIV-1 replication in macrophages can be modulated by a variety of inflammatory mediators and cytokines [23]–[25], identifying factors that influence HIV-1 growth in these cells is essential to understand the immunopathogenesis of HIV-1 infection and to design novel strategies to control HIV-1 propagation. We recently reported that the neuroimmunomodulatory molecule Nerve Growth Factor (NGF) stimulates HIV-1 replication in primary monocyte-derived macrophages [26], and we now address whether the immunosuppressive neuropeptides VIP and PACAP, which also regulate the functioning of the neuro-immune-endocrine system, could also affect HIV-1 production in those cells.

Few studies have addressed the biological effects of VIP and PACAP during HIV-1 infection, which have mainly focused on the repercussion of VIP and PACAP receptor ligation on HIV-1 production, describing that VPAC1 facilitates productive HIV-1 infection in CD4^+^ T cell lines [27], and that VPAC2 stimulation diminishes HIV-1 production in peripheral blood mononuclear cells (PBMCs) and in CD4^+^ T cell lines [28]. These findings suggest that the sole activation of VPAC1 or VPAC2 receptors can lead to opposite effects on HIV-1 replication, but the consequence of the simultaneous ligation of these receptors and PAC1 by their natural ligands on viral production is unknown. Therefore, because the existent data regarding the influence of VIP and PACAP receptors on HIV-1 infection were obtained in T cells using selective receptor agonists, we analyzed whether these neuropeptides could directly modulate the viral production in HIV-1-infected monocyte-derived macrophages, a possibility that has not been pursued thus far. We found that VIP and PACAP increased macrophage resistance to HIV-1 replication by inducing the synthesis of β-chemokines and IL-10 following preferential activation of the receptors VPAC2 and PAC1.

## Materials and Methods

### Ethics Statement

All experimental procedures involving human cells were performed with samples obtained after written informed consent and were approved by the Research Ethics Committee of the Oswaldo Cruz Foundation/Fiocruz (Rio de Janeiro, RJ, Brazil) under the number 397-07.

### HIV-1 isolates and reagents

The CCR5-dependent isolate HIV-1_Ba-L_ was obtained through the AIDS Research and Reference Reagent Program (NIH, Bethesda, MD). The neuropeptides VIP and PACAP and the VIP antagonist, which blocks both the VPAC1 and VPAC2 receptors, were from Anaspec (USA). The recombinant protein Maxadilan (PAC1 agonist) and its truncated form MaxadilanΔ65 (M65; PAC1 antagonist) were kindly donated by Dr. Ethan A. Lerner (Department of Dermatology, Massachusetts General Hospital, MA, USA). The VPAC1 and VPAC2 agonists Ala^11,22,28^-VIP and Bay 55-9837, respectively, were obtained from Tocris Bioscience (Bristol, UK). The neutralizing antibodies to CCL3, CCL4 and CCL5 and to the IL-10 receptor were obtained from Peprotech (NJ, USA) and Abcam (MA, USA), respectively. The endotoxin levels in the VIP and PACAP preparations were below the lower limit of detection (0.1 EU/mL), as measured by the Limulus Amebocyte Lysate (LAL) assay (Lonza).

### Cells

Human monocyte-derived macrophages were obtained from PBMCs that had been isolated by density gradient centrifugation (Ficoll-Paque Premium 1.077; GE Healthcare Biosciences, PA, USA) from buffy coat preparations of blood from healthy donors, through adherence onto plastic plates. Briefly, 1.5 – 2.0×10^6^ PBMCs were plated onto 48-well plates (Corning, MA, USA) in Dulbecco’s modified Eagle’s medium (DMEM; LGC Bio, SP, Brazil) containing 10% normal human serum (EMD Millipore, MA, USA) and penicillin-streptomycin (LGC Bio, SP, Brazil). Cells were maintained at 37°C in 5% CO_2_ for 6–7 days for monocyte differentiation into macrophages. Non-adherent cells were washed out, and the remaining macrophage layer was maintained in DMEM with 5% human serum. Macrophage purity was > 90%, as determined by flow cytometry (FACScan; Becton Dickinson, NJ, USA) analysis using anti-CD3 (BD Biosciences Pharmingen, CA, USA) and anti-CD68 (Southern Biotech, AL, USA) monoclonal antibodies.

### Macrophage production of β-chemokines and IL-10

Uninfected macrophages were treated with VIP or PACAP (10 nM), and concentrations of the β-chemokines CCL3 and CCL5 and of the cytokine IL-10 in the culture supernatants were measured using specific ELISA kits (R&D Systems, MN, USA, and eBioscience Inc, CA, USA, respectively). The results are shown as mass/volume and also by the area under curve (AUC) transformation, which allows a global analysis of the induced production of the mediators.

### HIV-1 infection

Macrophages were exposed for 16–18 h to viral suspensions containing 5–10 ng/mL of HIV-1 p24 antigen, as previously described [29]. The infected cells were then washed, replenished with fresh medium and maintained under standard culture conditions. HIV-1 replication was evaluated in cell culture supernatants after 12–14 days using an ELISA kit for HIV-1 p24 antigen (ZeptoMetrix Corp, NY, USA). Due to the common donor-to-donor variation of HIV-1 replication in primary cells [30], some HIV-1 inhibition results are presented normalized to viral production by macrophages maintained only in culture medium, with the absolute values shown in the legend of the figure.

### Effect of VIP and PACAP on HIV-1 replication

HIV-1-infected macrophages were treated either with VIP or PACAP immediately after cell infection, and viral production was measured as described above. After establishing that VIP and PACAP decreased viral replication, we addressed the relative contributions of the VIP and PACAP receptors using two different approaches. Initially, acutely HIV-1-infected cells were exposed to receptor antagonists for 15 min followed by the addition of VIP and PACAP to cell preparations. In later experiments, infected macrophages were treated with specific pharmacological agonists of the VIP and PACAP receptors (as listed above). In another set of experiments, HIV-1-infected macrophages were treated with either neuropeptide five days after infection together with neutralizing antibodies to the IL-10 receptor (1 µg/mL) or to the β-chemokines CCL3, CCL4 and CCL5 (1 µg/mL each). HIV-1 replication was evaluated as previously described.

### Statistical Analysis

All results presented in this study were prepared using GraphPad Prism 5.0 software (CA, USA). Statistical analysis calculation was performed using one-way ANOVA and the Tukey-Kramer tests. The results are shown as the mean ± SEM (standard error of the mean), and the comparisons between values were considered significantly different when the *p* value was less than 0.05 (*p*≤.05  =  *; *p*≤.01  =  **; *p*≤.001  =  ***).

## Results

### VIP and PACAP treatment inhibited HIV-1 production in macrophages

**Figure 1:**
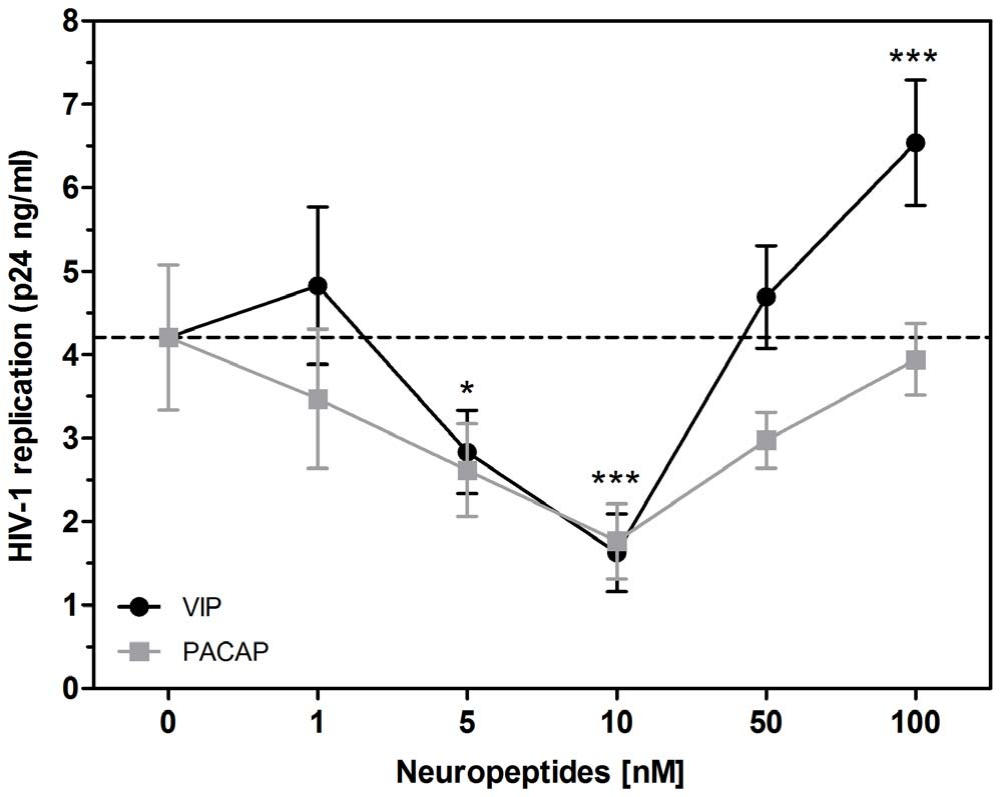
VIP and PACAP inhibit HIV-1 replication . Macrophages were infected with an R5-tropic HIV-1 isolate (Ba-L) and treated once with different concentrations of the neuropeptides, as indicated. Virus replication was measured in the culture supernatants by an HIV-1 p24 ELISA 12-14 days after infection. Data represent means ± SEM of five independent experiments for each peptide. *p≤.05; ***p≤.001.

Because activation of the receptors VPAC1 and VPAC2 has previously resulted in opposite effects during HIV-1 infection [27], [28], we initially investigated whether the neuropeptides VIP and PACAP, the natural ligands of those receptors, would also affect HIV-1 replication. To test this hypothesis, HIV-1-infected monocyte-derived macrophages were treated with VIP or PACAP. We first observed that both neuropeptides induced a significant reduction in virus replication (Fig. 1). VIP and PACAP were each individually able to decrease HIV-1 replication, achieving 33% and 38% of viral inhibition at 5 nM and 62% and 58% at 10 nM concentrations for VIP and PACAP, respectively. These results suggest that both neuropeptides were similarly effective in their ability to reduce HIV-1 production in macrophages. Higher concentrations of VIP or PACAP did not inhibit virus production and actually enhanced it (VIP at 100 nM), possibly due to receptor desensitization or an inverse agonist effect, as discussed later. Therefore, the next experiments were performed using the optimal inhibitory concentration of 10 nM for both neuropeptides.

### VIP and PACAP present synergistic and additive effects on HIV-1 inhibition

**Figure 2:**
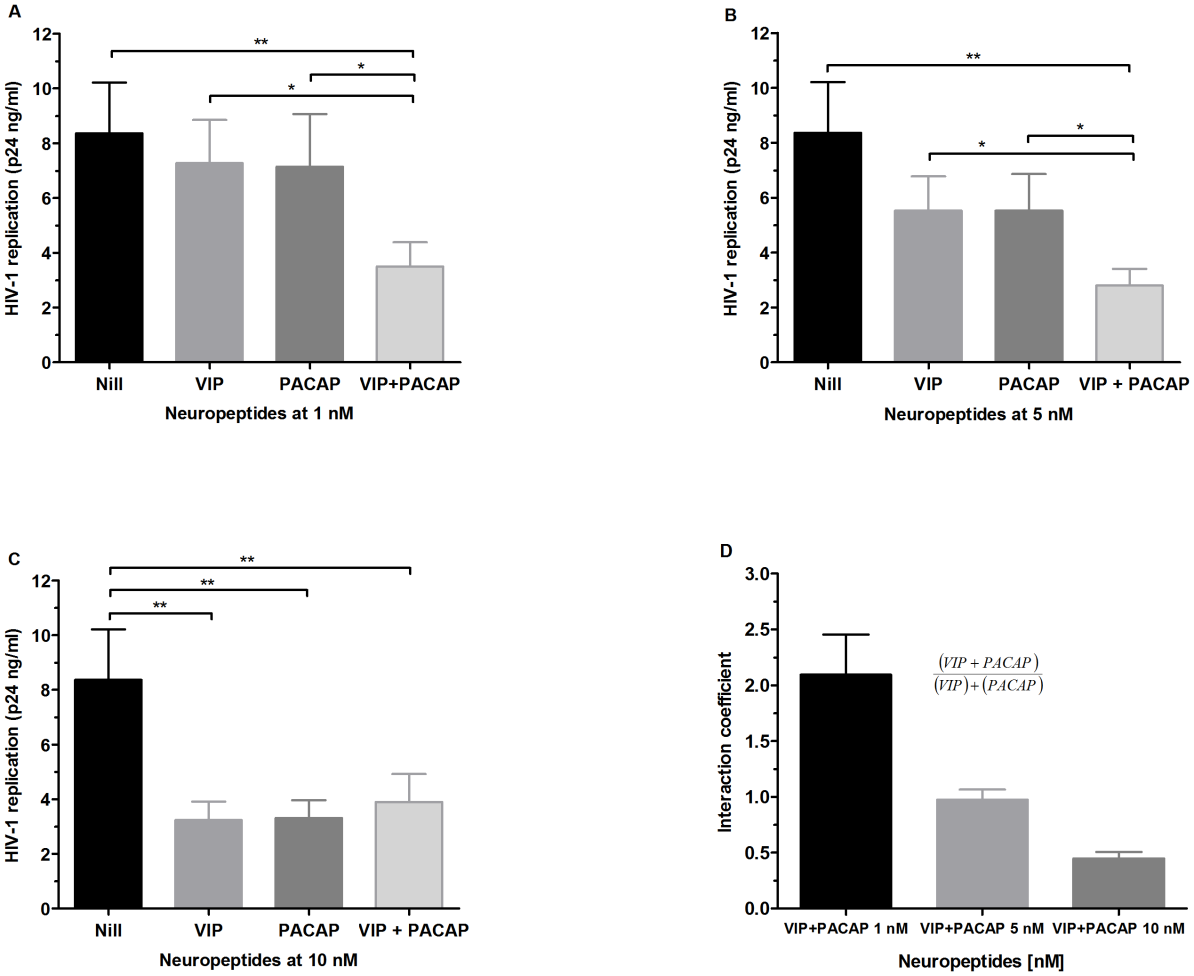
Effects of combined VIP and PACAP treatment on HIV replication . HIV-1-infected macrophages were simultaneously treated with 1 nM, 5 nM or 10 nM of VIP and PACAP (A, B and C, respectively), and virus replication was measured as above. Data represent means ± SEM of three independent experiments. (D) Equation used to calculate the interaction coefficient of VIP and PACAP at the indicated concentrations, based on the levels of HIV-1 inhibition shown in A, B and C. *p≤.05; **p≤.01; ***p≤.001.

As VIP and PACAP share receptors, we analyzed whether these neuropeptides could cooperatively modulate HIV-1 replication by exposing infected macrophages to combinations of sub-optimal or optimal viral inhibitory concentrations of VIP and PACAP. Combinations of 1 nM and 5 nM significantly potentiated inhibition relative to their individual activities, while no increment of HIV-1 inhibition occurred when both peptides were combined at a concentration of 10 nM (Fig. 2). To accurately classify the nature of this finding, we calculated the interaction coefficient of VIP and PACAP at those concentrations by dividing the inhibition percentages found when the peptides were associated by the sum of the inhibition of each isolated peptide (Fig. 2D; an interaction coefficient on the order of 1 indicates an additive phenomenon, whereas values greater than 1 indicate a synergistic effect). Therefore, VIP and PACAP synergize at 1 nM and act in an additive manner on viral production at 5 nM. These results suggest that combinations of small concentrations of VIP and PACAP could result in potent pharmacological activity, which could be physiologically relevant in situations where both peptides are simultaneously present in the tissue microenvironment.

### Receptor engagement in the VIP and PACAP modulation of HIV-1 replication

**Figure 3:**
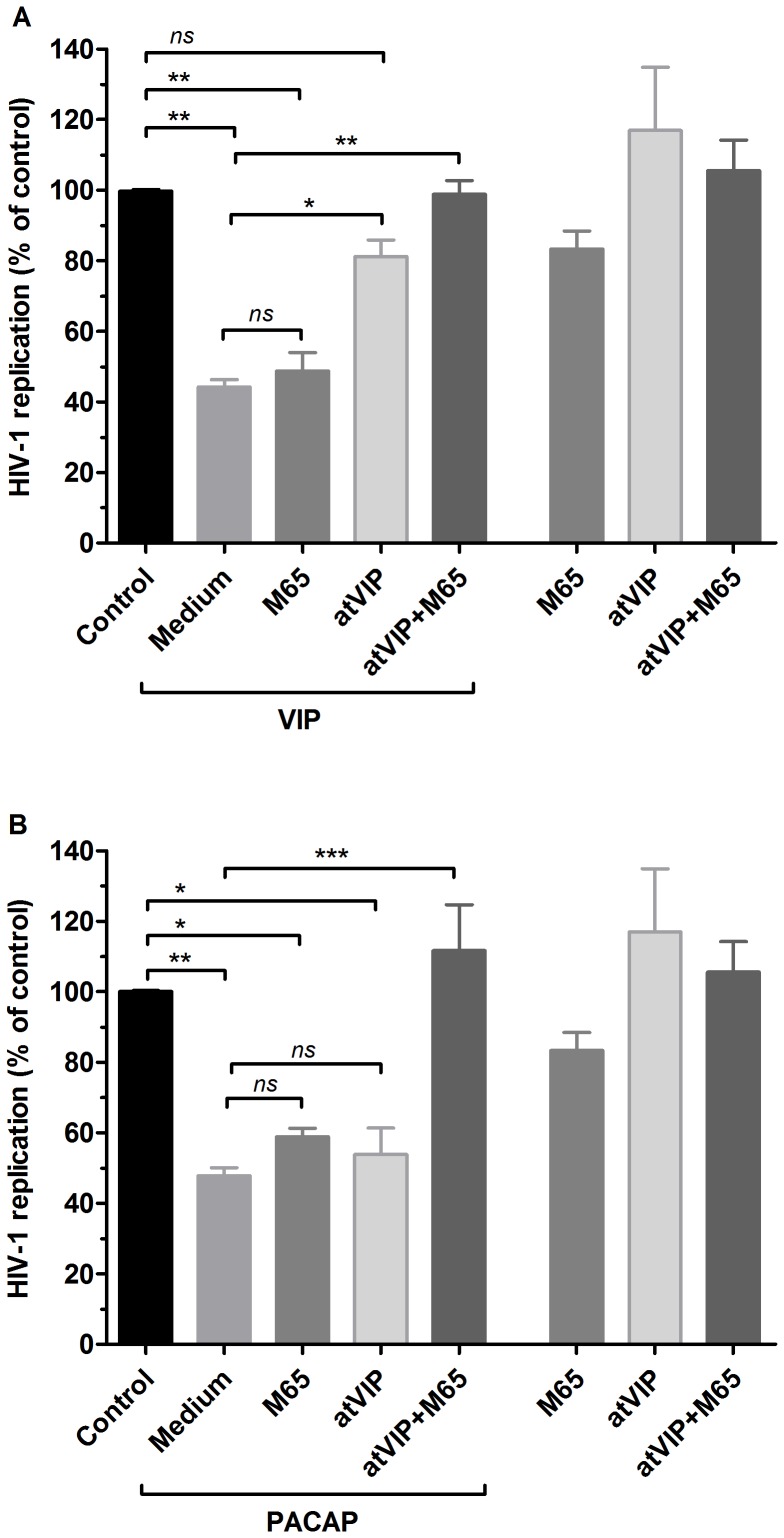
Contribution of VIP and PACAP receptors for the neuropeptide-induced inhibition of HIV-1 replication . Macrophages were infected with an R5-tropic HIV-1 isolate (Ba-L) and treated with culture medium (*Medium*) or with an antagonist of PAC1 (*M65*, 50 nM), VPAC1 and VPAC2 (*atVIP,* 100 nM) or with both antagonists (*M65+atVIP*) 15 minutes before the addition of VIP (A) or PACAP (B) at 10 nM. Viral replication was measured in the culture supernatants using an HIV-1 p24 ELISA 12-14 days after infection. Data represent means ± SEM of five independent experiments. Virus production in the positive control (HIV-1-infected cells cultured only with medium): 5.8±1.9 ng/mL p24 Ag. The three bars on the right show the virus replication by macrophages exposed only to the antagonists. *p≤.05; **p≤.01; ***p≤.001.

Because VIP preferentially activates the VPAC1 and VPAC2 receptors and PACAP binds the three receptors with high affinity [5]–[8], we analyzed the neuropeptide dependence of these receptors to inhibit HIV-1 production using two distinct assays. Initially, we added specific antagonists of PAC1 or VPAC1/2 to HIV-1-infected cells before treating them with VIP or PACAP. As shown in Fig 3A, VIP-induced HIV-1 inhibition is largely dependent on VPAC1/2, since blockade of both receptors abrogated the VIP-mediated inhibition of HIV-1 production with no significant changes following PAC1 blockade. We also observed that PACAP could inhibit HIV-1 replication via activation of all three receptors, as its ability to decrease viral growth was maintained when VPAC1/2 or PAC1 was antagonized separately but was abolished when all three receptors were blocked together (Fig 3B).

**Figure 4:**
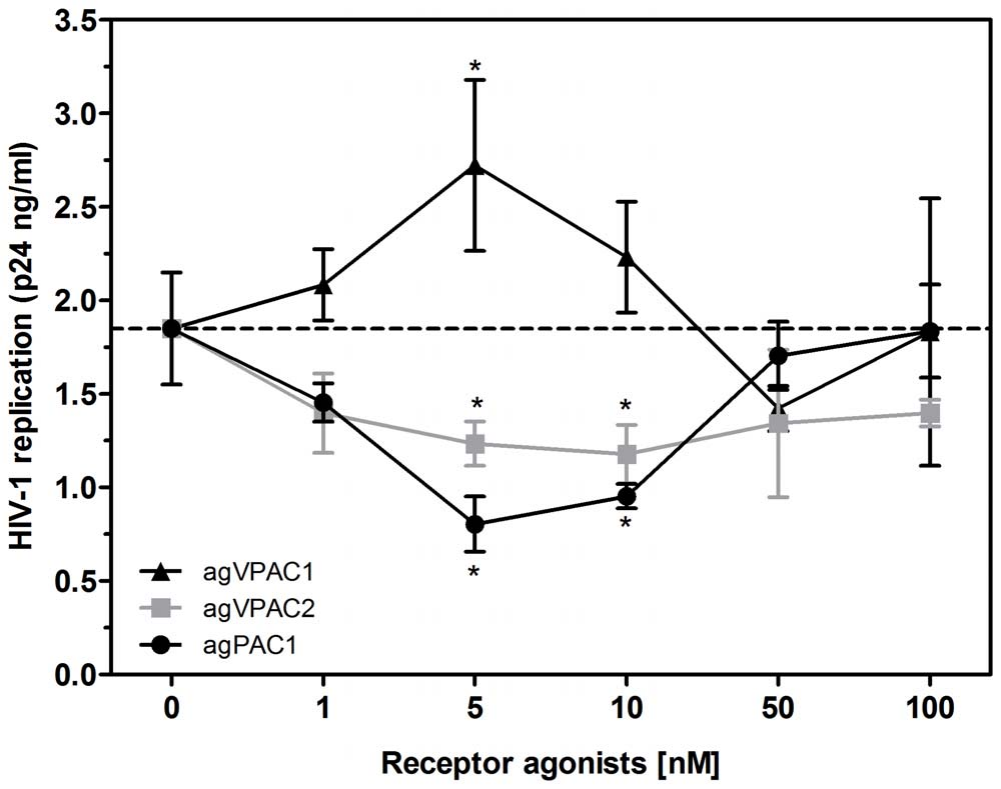
Specific activity of VIP and PACAP receptors on HIV-1 replication . Macrophages were infected with an R5-tropic HIV-1 isolate (Ba-L) and treated once with different concentrations of agonists for the VPAC1 (*agVPAC1*), VPAC2 (*agVPAC2*) or PAC1 (*agPAC1*) receptors, as indicated, and viral replication was measured in the culture supernatants using an HIV-1 p24 ELISA 12-14 days after infection. Data represent means ± SEM of four independent experiments. *p≤.05.

We further studied the role of the individual receptors in the VIP- and PACAP-mediated regulation of HIV-1 replication by using specific agonists to VPAC1, VPAC2 and PAC1 (ALA-VIP, Bay 55 and Maxadilan, respectively). We found that the VPAC1 agonist at 5 nM increased HIV-1 production by 48%, whereas the VPAC2 agonist at 5 nM and 10 nM reduced viral growth by 31% and 35%, respectively. The PAC1 agonist at 5 nM and 10 nM decreased HIV-1 replication by 56% and 46%, respectively (Fig 4). Of note, the optimal concentrations of the receptor agonists that significantly modulated viral production were similar to those of the natural receptor ligands VIP and PACAP.

**Figure 5:**
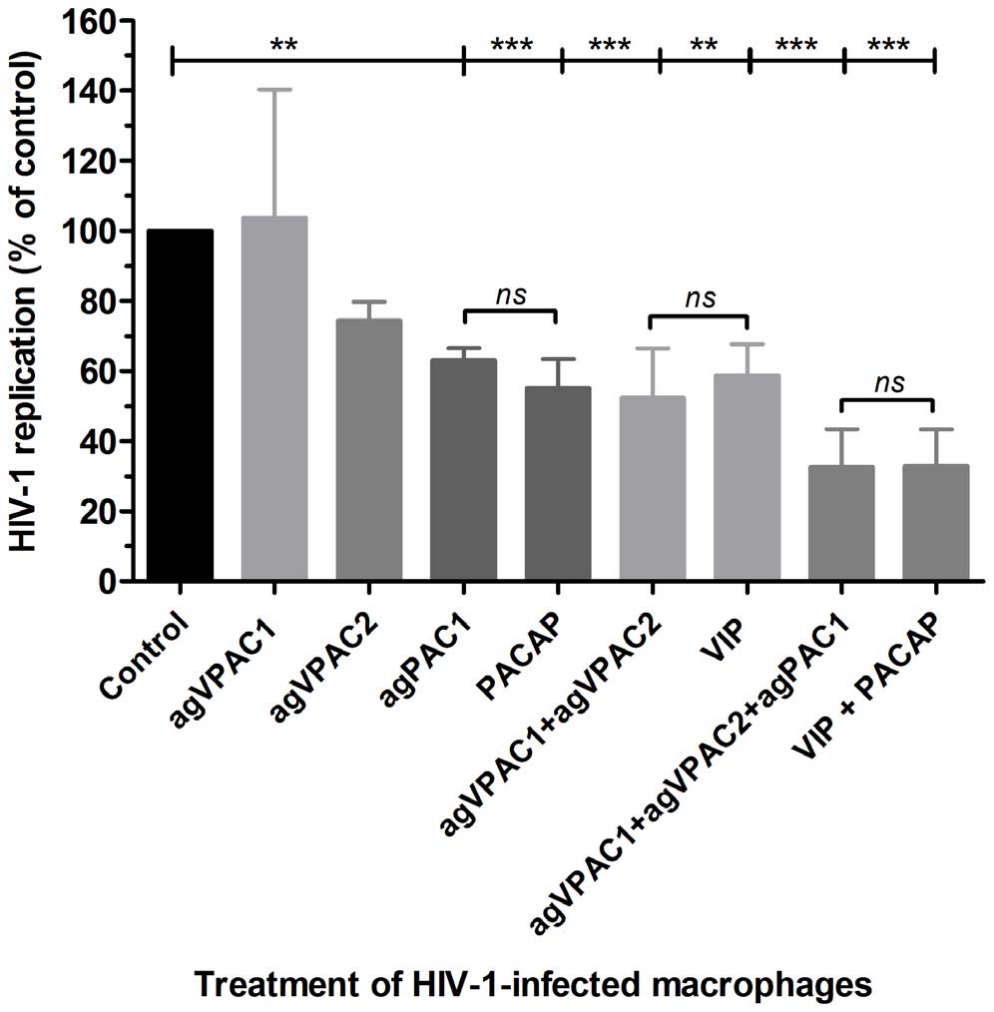
Combined use of receptor agonists reproduces VIP and PACAP effects on HIV-1 replication . Macrophages were infected with an R5-tropic HIV-1 isolate (Ba-L) and treated with agonists for the VPAC1 (*agVPAC1* 2.5 nM), VPAC2 (*agVPAC2* 2.5 nM) or PAC1 (*agPAC1*, 5 nM) receptors or with VIP (5 nM) and PACAP (5 nM), either alone or in combination, as indicated. Viral replication was measured in the culture supernatants using an HIV-1 p24 ELISA 12–14 days after infection. Viral production in the positive control (HIV-1-infected cells cultured only with medium): 3.0±0.8 ng/mL p24 Ag. **p≤.01; ***p≤.001.

Due to the opposing effects of VPAC1 and VPAC2 or PAC1 engagement, we analyzed the outcome of agonist combinations on viral production. For these assays we used the agonists at sub-optimal concentrations to allow for comparison with non-saturating doses of the natural ligands VIP and PACAP. Combination treatment with VPAC1 and VPAC2 agonists decreased HIV-1 replication by 48%, similar to the effect observed with sub-optimal doses of VIP. Their combined usage with a PAC1 agonist reduced viral growth by 68%, similar to the additive effects observed during co-treatment with VIP and PACAP (Fig. 5). These results showed that combination treatment with agonists of the different receptors mimicked the receptor preference of both natural peptides in reproducing the individual or additive effects of VIP and PACAP treatment on HIV-1 replication. Of note, simultaneous activation of VPAC1 and PAC1 slightly increased HIV-1 replication, whereas binding of VPAC2 plus PAC1 did not change HIV-1 replication (data not shown). In conclusion, VIP depended on the ligation of VPAC1/2 to increase macrophage resistance to HIV-1 growth, and PACAP promoted the same phenomenon either by activating PAC1 only or through activating VPAC1/2 plus PAC1.

### VIP and PACAP increase β-chemokine production by macrophages

**Figure 6:**
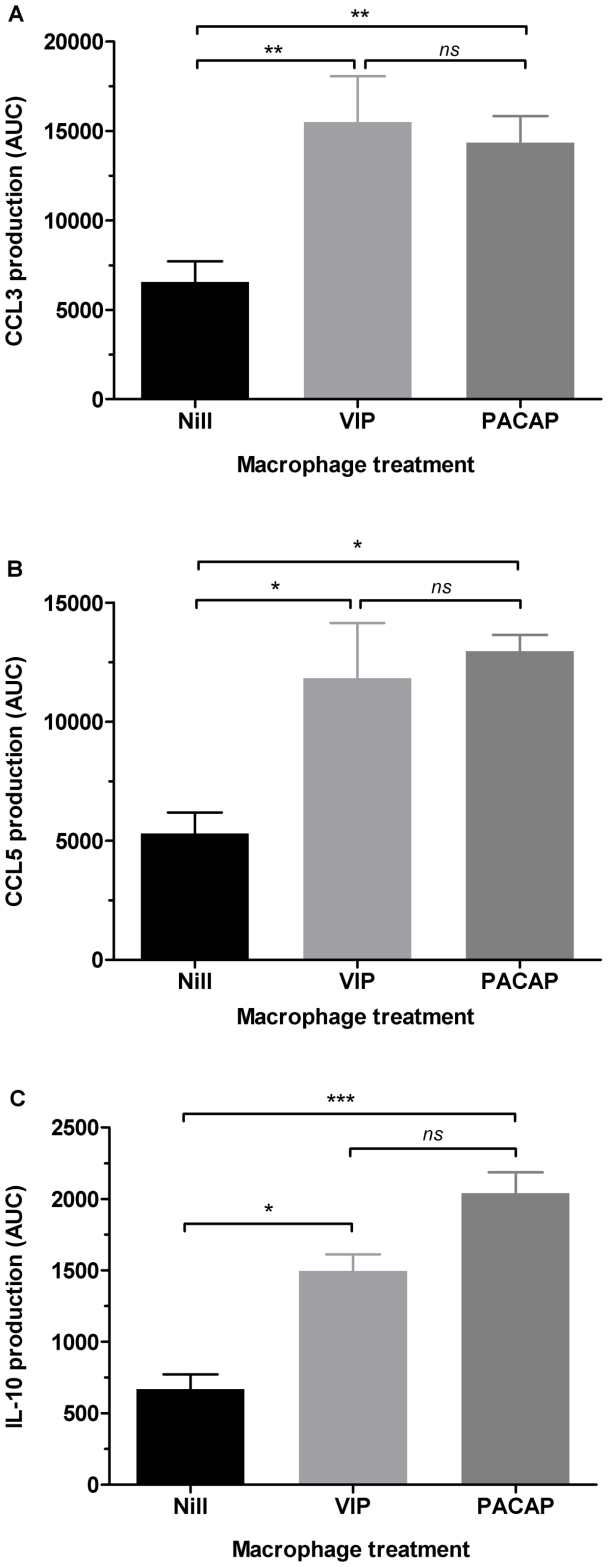
VIP and PACAP induce CCL3, CCL5 and IL-10 production in macrophages . Figure shows the production of CCL3 (A), CCL5 (B) and IL-10 (C) by area under the curve (AUC) analysis, which was calculated based on the respective concentrations measured by ELISA (See Figure S1). Data represent means ± SEM of six (CCL3) and four (CCL5 and IL10) independent experiments. *p≤.05; **p≤.01; ***p≤.001.

VIP and PACAP can induce the production of the β-chemokines CCL3, CCL4 and CCL5 in microglial cells, an effect associated with the prevention of HIV-1 gp120-induced apoptosis [31], [32]. Because β-chemokines are potent inhibitors of HIV-1 infection [33], [34], we evaluated whether VIP and PACAP could also induce macrophage secretion of these molecules. Indeed, both neuropeptides enhanced macrophage release of CCL3 and CCL5, with CCL3 production peaking 96 h after stimulation with either peptide (Fig. S1A) and the maximum production of CCL5 occurring 24 h and 48 h after VIP or PACAP stimulation, respectively (Fig. S1B). VIP and PACAP more than doubled CCL3 and CCL5 production relative to untreated cells based on measuring chemokine production by the area under the curve (AUC) (Figs. 6A and 6B). Moreover, because the differences in chemokine production induced by VIP or PACAP were not significantly different (based on AUC), we can postulate that the ability of either peptide to induce chemokine production is similar.

### VIP and PACAP increase IL-10 production by macrophages

The immunomodulatory activities of VIP and PACAP are at least partially dependent of IL-10 [9], an anti-inflammatory cytokine able to inhibit the HIV-1 replication [35], [36]. We also detected that VIP and PACAP increase macrophage production of IL-10 (Fig. S1C), which peaked 48 h after the stimuli. Based on AUC analyses (Fig. 6C), VIP treatment doubled IL-10 production, whereas PACAP treatment tripled macrophage release of this cytokine.

### Contribution of β-chemokines and IL-10 to the VIP- and PACAP- mediated inhibition of HIV-1 replication

**Figure 7:**
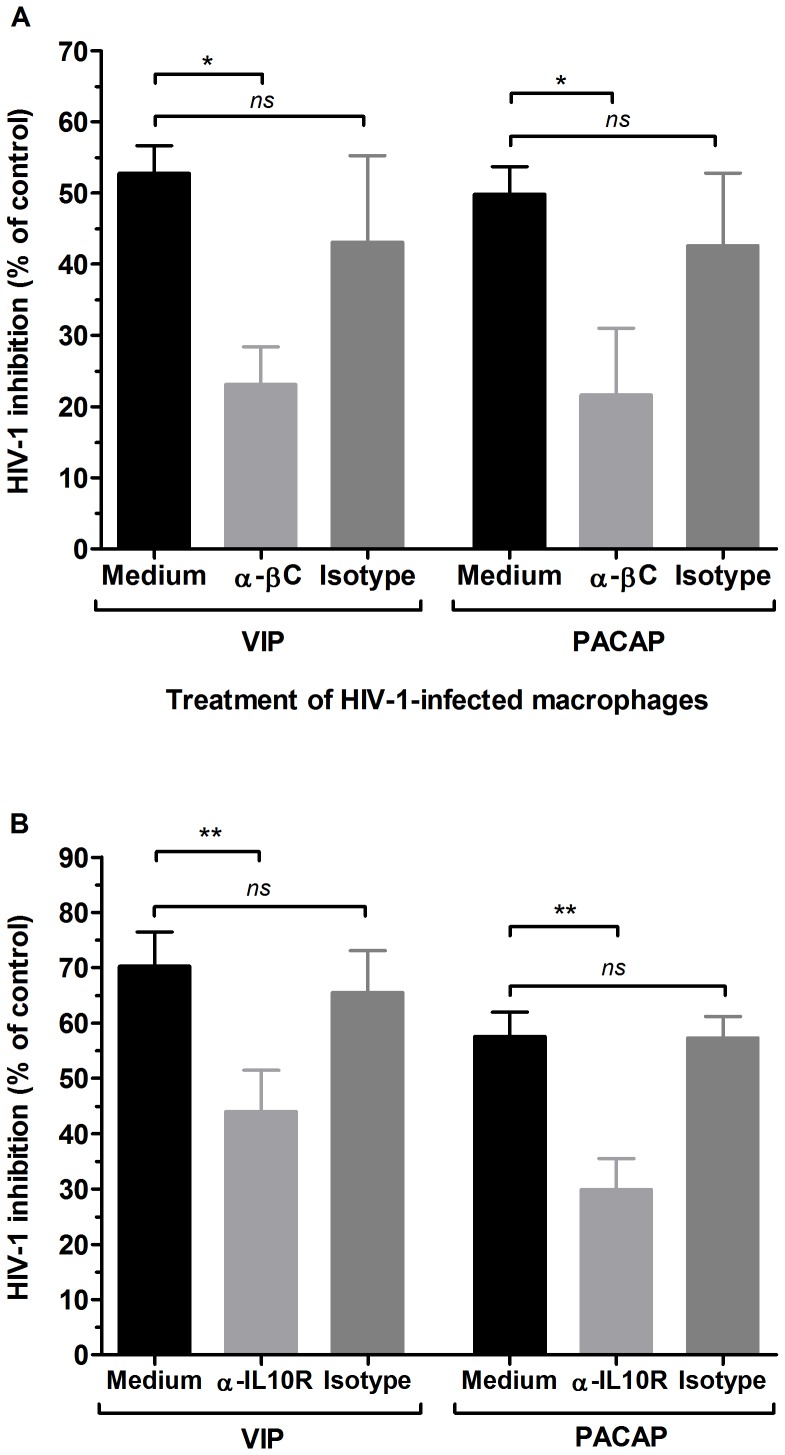
β-chemokines and IL-10 are implicated in the VIP- and PACAP-induced inhibition of HIV-1 replication . Macrophages were infected with an R5-tropic HIV-1 isolate (Ba-L), and after 5 days, were treated with VIP or PACAP plus anti-CCL3, CCL4 and CCL5 antibodies (*α-βC)* (A), anti-IL-10 receptor antibodies (*α-IL10R*), or isotype control antibodies. Viral replication was measured in the culture supernatants using an HIV-1 p24 ELISA 12–14 days after infection. Data represent means ± SEM of five (A) or four (B) independent experiments. Virus production in the positive control (HIV-1-infected cells cultured only with medium): (A) 14.8±9.0 ng/mL and (B) 14.5±7.0 ng/mL p24 Ag. *p≤.05; **p≤.01.

We next evaluated whether these mediators were implicated in the ability of VIP and PACAP to reduce HIV-1 growth by adding the neuropeptides to infected macrophages together with neutralizing antibodies to CCL3, CCL4 and CCL5, or to the IL-10 receptor. This experiment was conducted five days after infection because this is the approximate time when the infection becomes productive, thus allowing the antibodies to neutralize the anti-HIV-1 effector molecules when new rounds of infections were occurring. Indeed, neutralization of those three β-chemokines and blocking of the IL-10 receptor significantly reduced the inhibitory effects of VIP and PACAP on HIV-1 replication (Fig. 7), showing that CCL3, CCL4 and CCL5 and IL-10 are implicated in neuropeptide-mediated inhibition of HIV-1 growth.

## Discussion

VIP and PACAP are pleiotropic factors associated with a number of physiological processes, such as endocrine, metabolic and gastrointestinal effects, also including modulatory effects on the immune system. The receptors for VIP and PACAP, the G coupled-receptors VPAC1, VPAC2 and PAC1, are widely distributed, a feature that allows VIP and PACAP to exert their large range of effects. Here, we show that VIP and PACAP treatment increased resistance to HIV-1 replication in primary macrophages by inducing the production of the β-chemokines CCL3 and CCL5 and the cytokine IL-10, molecules that are able to reduce HIV-1 growth in vitro. Our study focused, for the first time, on the ability of the natural peptides VIP and PACAP to modulate HIV-1 infection in a primary target cell, in addition to defining the relative contribution of each of their receptors to this phenomenon. These findings have clear implications in the understanding of the role of VIP and PACAP in the pathogenesis of HIV-1 infection.

Treating HIV-1-infected macrophages with these naturally occurring neuropeptides diminished viral production, and treatment with specific agonists of the neuropeptide receptors VPAC2 and PAC1 showed similar effects. The inhibitory effect on viral replication was not dose-dependent within the range of tested concentrations of VIP and PACAP between 1 nM and 100 nM, with the optimal inhibitory concentration at 10 nM for both peptides. The finding that VIP treatment at a concentration 5-fold that of the optimal HIV-1 inhibitory concentration and PACAP treatment at a 10-fold ratio did not influence viral production may be explained by receptor desensitization. On the other hand, the observed increase in HIV-1 production induced by 100 nM VIP could be explained by the property of inverse agonism that occurs for some ligands at saturating concentrations, a phenomenon that has already been described for a variety of G-coupled receptor ligands [37]. Alternatively, taking into account that the sole activation of VPAC1 facilitates HIV-1 infection in CD4^+^ T cell lines (27), one could explain the HIV-1 promoting effect by VIP at 100 nM due to a possible preferential engagement of VPAC1 at high VIP concentrations, although it has been described that the affinity of VIP for its receptors VPAC1 and VPAC2 is similar [4]–[7]. Importantly, the concentration of both neuropeptides that effectively inhibited HIV-1 production (10 nM) also possesses a variety of immunomodulatory roles [38]–[41], suggesting that the ability of these peptides to influence HIV-1 replication in macrophages is associated with their immunoregulatory activities on these cells.

The ability of VIP and PACAP to down-regulate HIV-1 production became even more evident in light of the experiment testing their additive and synergistic activity on infected macrophages. Both neuropeptides presented an additive effect at 5 nM concentration, and showed potent synergy at 1 nM concentration, and their combined effects at these concentrations were the same as those observed following treatment with each molecule individually at 10 nM concentration (Fig. 2). Furthermore, the addition of sub-optimal concentrations of the receptor agonists to HIV-1-infected macrophages restricted the viral growth in a similar manner to treatment with equivalent doses of VIP and PACAP (Fig. 5). Thus, even taking into account that the activation of the VPAC1 receptor may favor HIV-1 replication, we believe that the concomitant engagement of the three receptors by the native neuropeptides, as presumably occurs in lymphoid tissues, will indeed enhance the macrophage resistance to HIV-1 growth.

VIP promoted HIV-1 inhibition through stimulation of the receptors VPAC1 and VPAC2 but not through stimulation of PAC1. This effect likely occurred due to the high affinity of VIP for VPAC1 and VPAC2 and its low affinity for PAC1. The ability of PACAP to diminish HIV-1 replication, on the other hand, resulted from its ligation of all three receptors because its effect was only abrogated when all three receptors were blocked. Because the PACAP affinity for PAC1 is higher than its affinity for VPAC1 or VPAC2 [8], it is conceivable that it inhibits HIV-1 upon preferential binding to PAC1, but can also exert an inhibitory effect following ligation to VPAC1 and VPAC2 in the absence or hindrance of PAC1, or in a situation of excess PACAP concentration.

We defined the receptors preference for the HIV-1 inhibitory activity of VIP and PACAP using specific receptor agonists. The combination of these molecules not only mimicked the effects of the natural neuropeptides but also established the receptors VPAC2 and PAC1 as HIV-1 inhibitory mediators, and VPAC1 as an enhancer of HIV-1 production. Our findings are in agreement with previous studies that reported that VPAC1 facilitates productive HIV-1 infection in CD4^+^ tumor cell lines [27], and that VPAC2 activation inhibits HIV-1 integration and viral production in CD4^+^ tumor cell lines and PBMCs [28]. In addition, we defined the role of the PAC1 receptor during HIV-1 infection. Therefore, it is reasonable to assume that the simultaneous activation of these receptors by their natural ligands VIP and PACAP results in reduced HIV-1 replication in macrophages, even under conditions in which the receptor VPAC1 is present and activated, and that the HIV-1-enhancing activity of the sole activation of VPAC1 activation is overcome by the concomitant recruitment of the inhibitory receptors VPAC2 and PAC1.

VIP and PACAP induced macrophage release of CCL3 and CCL5, which contributed to the HIV-1 inhibitory effect elicited by both neuropeptides as evidenced by immunoneutralization of both β-chemokines (and CCL4 as well). VIP and PACAP can inhibit β-chemokine production upon pro-inflammatory induction [40], [42], but they are also able to stimulate the production of chemokines in different settings [31], [32], [43]–[45], pointing out their ability to regulate the chemokine axis. VIP and PACAP have been suggested to confer a protective role against HIV-1 in the central nervous system through CCL3 and CCL5 production [46], and our results now imply that these neuropeptides can offer a systemic protection against HIV-1 by boosting macrophage resistance to viral growth by augmenting the secretion of these mediators.

A concurrent mechanism implicated in HIV-1 inhibition by VIP and PACAP is their ability to induce macrophage secretion of IL-10, a cytokine that inhibits HIV-1 by interfering in the reverse transcription of viral RNA [35], [47]. IL-10 is readily produced by human and murine cells stimulated by VIP and PACAP and is a critical mediator of the anti-inflammatory properties of these neuropeptides. Here, we found thatIL-10 neutralization reduced the ability of VIP and PACAP to inhibit HIV-1 replication, indicating that IL-10, likewise the β-chemokines is required to increase macrophage resistance following treatment with these neuropeptides.

In conclusion, our study shows that VIP and PACAP, molecules endowed with a large spectrum of physiological activities on the neuro-immune-endocrine system, can increase macrophage resistance to HIV-1 replication. It is remarkable to observe the contrasting roles of neurotrophic molecules on the HIV-1 biology, as we recently showed that Nerve Growth Factor (NGF) favors viral replication in macrophages [26]. Our findings also open new possibilities for therapeutic strategies, in which HIV-1 replication could be controlled through activation of VPAC2 and PAC1 receptors through their natural ligands VIP and PACAP, or by their specific agonists, in association with the present available antiretroviral compounds.

## Supporting Information

Figure S1 **VIP and PACAP induce CCL3, CCL5 and IL-10 production in macrophages**. Macrophages were left untreated (*Nil)* or treated with VIP or PACAP (10 nM), and supernatants were collected at different time-points. The concentrations of CCL3 (A), CCL5 (B) and IL-10 (C) were measured by ELISA. Data represent means ± SEM of six (CCL3) and four (CCL5 and IL10) independent experiments.
(TIF)
